# Designing a national network of pancreas units: the Italian model for high-quality pancreatic cancer care

**DOI:** 10.1007/s13304-026-02598-7

**Published:** 2026-03-24

**Authors:** Sergio Alfieri, Giuseppe Quero, Vincenzo Tondolo, Giampaolo Balzano, Antonella Cardone, Giovanni Conzo, Carlo Fabbri, Viviana Ferrari, Luca Frulloni, Salvatore Gruttadauria, Elisabetta Ianelli, Evaristo Maiello, Roberta Menghi, Michele Milella, Michele Reni, Roberto Salvia, Cristiano Spada, Manuela Tamburo De Bella, Ilaria Tarantino, Giampaolo Tortora, Federica Valsecchi, Leonardo Vincenti, Fabio Vistoli, Ugo Boggi

**Affiliations:** 1 Pancreatic Surgery Unit, Fondazione Policlinico Universitario “Agostino Gemelli” IRCCS, Rome, Italy; 2 Dipartimento di Scienze Mediche e Chirurgiche, Università Cattolica del Sacro Cuore di Roma, Rome, Italy; 3 Gemelli Pancreatic Center, CRMPG (Advanced Pancreatic Research Center) Fondazione Policlinico Universitario “Agostino Gemelli” IRCCS, Rome, Italy; 4 General Surgery Unit, Fatebenefratelli Isola Tiberina—Gemelli Isola, Rome, Italy; 5 Division of Pancreatic Surgery, Circolo di Varese Hospital, Varese, Italy; 6 Cancer Patients Europe, Brussels, Belgium; 7 Division of General and Oncological Surgery, Department of Translational Medical Sciences, University of Campania ‘Luigi Vanvitelli’, Naples, Italy; 8 Digestive Endoscopy and Gastroenterology Unit, Forlì-Cesena Hospitals, Azienda Unita Sanitaria Locale della Romagna, Forlì-Cesena, Italy; 9 Nastro Viola Association, Bergamo, Italy; 10 Department of Medicine, Gastroenterology and Digestive Endoscopy, University of Verona, Verona, Italy; 11 Department for the Treatment and Study of Abdominal Diseases and Abdominal Transplantation, Istituto Di Ricovero E Cura a Carattere Scientifico-Istituto Mediterraneo Per I Trapianti E Terapie Ad Alta Specializzazione (IRCCS-ISMETT), Palermo, Italy; 12 Department of Surgery and Medical and Surgical Specialties, University of Catania, Catania, Italy; 13 Italian Federation of Volunteer-based Cancer Organizations (FAVO), Rome, Italy; 14 Medical Oncology Unit, Fondazione IRCCS “Casa Sollievo della Sofferenza”, San Giovanni Rotondo, Italy; 15 Section of Innovation Biomedicine-Oncology Area, Department of Engineering for Innovation Medicine, University of Verona, and Verona University and Hospital Trust, Verona, Italy; 16 Department of Medical Oncology, Pancreas Translational and Clinical Research Center, IRCCS San Raffaele Scientific Institute, Milan, Italy; 17 Department of Pancreatic Surgery, Azienda Ospedaliera Universitaria Verona, Verona, Italy; 18 Digestive Endoscopy Unit, Fondazione Policlinico Universitario Agostino Gemelli IRCCS, Rome, Italy; 19 Hospital Clinical Networks Governance e DM70/15 Monitoring—AGENAS—National Agency for Regional Health Services (Age.na.s), Rome, Italy; 20 Digestive Endoscopy Service, Department of Diagnostic and Therapeutic Services, IRCCS - ISMETT, Palermo, Italy; 21 Medical Oncology, Comprehensive Cancer Center, Fondazione Policlinico Universitario “A Gemelli”- IRCCS, Rome, Italy; 22 Nadia Valsecchi Foundation, Brescia, Italy; 23 Unit of Surgery, Department of Surgery Sciences, National Institute of Gastroenterology ““S. de Bellis”, IRCCS Research Hospital, Castellana Grotte, Bari, Italy; 24 Division of General and Transplant Surgery, San Salvatore Hospital ASL1 Abruzzo, L’Aquila, Italy; 25 Department of Biotechnological and Applied Clinical Sciences, University of L’Aquila, L’Aquila, Italy; 26 Division of General and Transplant Surgery, University of Pisa, Pisa, Italy; 27 Pancreatic Surgery Unit, Department of Surgery, Pancreatic Surgery Unit, Department of Surgery, Largo Agostino Gemelli, 8, 00168 Rome, Italy

**Keywords:** Pancreatic cancer, Hub-Spoke, Disease centralization, Multidisciplinary

## Abstract

Pancreatic and periampullary cancers are among the most challenging malignancies. Centralization of care and multidisciplinary management have been associated with improved outcomes. In 2024,the Italian Ministry of Health established a national Steering Committee with the objective of defining a centralized model for the management of pancreatic and periampullary diseases and identifying the minimum requirements for referral centers across Italy. The Steering Committee met every four weeks through plenary and focused working groups. Using data from the National Agency for Regional Health Services and the National Outcome Program, all ICD-9 codes related to pancreatic, periampullary, and distal biliary diseases were analyzed. Criteria were developed according to international recommendations and structured into a Hub-and-Spoke network model. Pancreas Units(Hub centers) were defined as hospitals providing 24/7 multidisciplinary expertise, advanced diagnostic and interventional capabilities, and a minimum surgical volume of ≥ 30 pancreatic resections per year with 90-day mortality < 10% (target < 5% within 3 years). Spoke centers were required to adhere to standardized diagnostic–therapeutic pathways and participate in multidisciplinary team meetings, without performing pancreatic surgery. Based on catchment area analyses, one Hub center per 700,000–1,200,000 inhabitants was recommended. Additional requirements included structured multidisciplinary processes, standardized diagnostic pathways, and integration with national research and training networks. This national model provides a comprehensive framework for centralizing pancreatic and periampullary diseases care. By defining minimum standards, enhancing multidisciplinary coordination, and concentrating complex procedures in high-performance centers, this initiative aims to improve outcomes, reduce regional disparities, and create a sustainable national Pancreas Unit network.

**Supplementary Information:**

The online version contains supplementary material available at 10.1007/s13304-026-02598-7.

## Introduction

Pancreatic cancer (PC) is currently the third leading cause of cancer-related mortality in Europe, with a reported 5-year survival rate of 12.5% and nearly 500,000 new cases diagnosed worldwide in 2021 [1].These data are expected to worsen substantially in the coming decades, with both incidence and mortality projected to increase by almost 35% over the next 30 years [1, 2].

Although surgery remains the cornerstone of PC treatment, only approximately 20% of patients are candidates for surgical resection, while the majority present with locally advanced or metastatic disease at diagnosis [3–5]. As a result, PC represents a complex condition that requires the coordinated involvement of multiple healthcare professionals for a comprehensive treatment at different stages [6, 7]. According to the Innovative Partnership for Action Against Cancer (IPAAC) [8], this process relies on two key elements: the implementation of a multidisciplinary approach and the centralization of diagnostic and therapeutic pathways in referral, high-volume centers. In this context, several studies have demonstrated a positive volume–outcome relationship in PC management, with improved short- and long-term outcomes in high-volume centers [9–11]. These findings further highlight the need to centralize PC care within experienced referral institutions. However, a universally accepted minimum threshold for defining a high-volume center for PC management is still lacking. While it is generally agreed that centralization requires substantial experience within high-performance hospital settings, the specific parameters that should define a referral center remain debated. This uncertainty stems partly from heterogeneous recommendations in the literature regarding the minimum number of pancreatic resections per year—currently the only widely measurable parameter besides operative mortality—and partly from the absence of clearly defined non-surgical criteria for identifying a pancreatic unit.

International scientific evidence has shown a strong association between hospital surgical volume for pancreatic procedures and operative mortality [12–14]. Similar trends have been observed in Italy: the risk of postoperative mortality is estimated to be five-fold higher in hospitals performing five or fewer pancreatic resections per year compared with those performing 90–100 procedures (operative mortality 12.4% vs. 2.6%) [15]. Nevertheless, many hospitals meeting apparently adequate minimum volume thresholds still report operative mortality rates exceeding 10%, and in some instances surpassing 40% [16]. This demonstrates that minimum surgical volume alone cannot serve as the sole criterion for selecting referral centers.

To date, only a few European countries have formally adopted a centralization model for PC care, with systems ranging from one highly specialized center per 2,000,000 inhabitants in the United Kingdom to one center per 900,000 inhabitants in the Netherlands [17].

Until now, Italy has lacked a national policy for the centralization of pancreatic and periampullary diseases care. Consequently, the quality of care has varied widely across regions and hospitals, contributing to substantial patient mobility toward specialized centers. Cross-regional mobility for PC treatment—particularly from southern to northern regions—has markedly increased in recent decades. Between 2014 and 2016, nearly 40% of patients from southern regions and 15% from central regions travelled to northern Italy for surgical treatment [16] (Online Resource 1). More recent data confirm the persistence of substantial interregional mobility, with cross-regional escape indices reaching up to 100% in 2022 in some regions, such as Molise and Valle d’Aosta, indicating the absence of locally available surgical pathways [16].reaching even higher value in 2022 with cross-mobility rates up to 100% in two regions (Molise and Valle d’Aosta) [16].

This raised significant concerns regarding equity of access, especially for patients with limited financial resources or with medical conditions that hinder long-distance travel.

Based on these considerations, a steering committee was established in 2024 by the Italian Ministry of Health to develop a national centralization model for the management of pancreatic and periampullary diseases. The aim of this study is, thus, to present the results of the steering committee regarding the centralization process for PC and periampullary diseases management and to summarize the requirements for the creation of referral centers.

## Methods

### Steering Committee

The Steering Committee was established in March 2024 by the Italian Ministry of Health and conducted its activities over a 12-month period. At the time of manuscript submission, the Committee had completed its mandate and delivered its final recommendations to the Ministry.

The Steering Committee comprised 25 specialists, including surgeons, oncologists, endoscopists, gastroenterologists, representatives of the Italian Ministry of Health, and delegates from patient associations involved in pancreatic cancer advocacy [18]. Meetings were held every four weeks and conducted both in person and remotely through plenary sessions and focused working groups at the Ministry of Health. Focused working groups addressed specific domains, including definition of minimum structural and organizational requirements for Pancreas Units, surgical volume and outcome metrics, multidisciplinary team composition and workflows, diagnostic and therapeutic pathways, training and workforce development, and integration within national research and registry frameworks.

The consensus process was based on an iterative, discussion-driven approach. Proposals were initially developed within focused working groups and subsequently discussed during plenary Steering Committee meetings. Consensus was achieved through progressive refinement of the proposals following structured discussion among all members, with final approval obtained by collegial agreement. When unanimity was not immediately reached, further discussion and revision were undertaken until a shared position was achieved. No formal voting or Delphi-like procedures were applied. All meeting-related documents, together with the collected literature and clinical practice guidelines for PC management, were stored in a shared online repository and regularly updated.

### Definition criteria for the treatment centralization

The data used to define the criteria for identifying referral centers for PC treatment were obtained from the Age.na.s. Age.na.s is a public agency of the Italian Ministry of Health, established in 1993 with the mandate to support regional administrations in ensuring equitable access to healthcare across the national territory. Since 2020, the agency has also been involved in strengthening both hospital-based and territorial healthcare networks to enhance the national response to epidemiological emergencies.

As part of its institutional activities, Age.na.s annually produces the National Outcome Program (Programma Nazionale Esiti, PNE), which measures, analyzes, evaluates, and monitors the clinical and healthcare performance of all medical facilities in Italy [16]. This program categorizes diseases and related procedures through the International Classification of Diseases, 9th Revision (ICD-9) codes and evaluates key clinical indicators, including disease-specific hospitalization rates, length of hospital stay, number of associated procedures, and 30- and 90-day mortality rates.

Given the absence of universally accepted criteria or volume thresholds in the literature to define referral centers for PC treatment, the Steering Committee relied on the data provided by Age.na.s and the PNE to develop the centralization criteria. All ICD-9 codes related to pancreatic cancer, periampullary diseases, distal bile duct diseases, and their corresponding treatment codes were included in the analysis. The decision to encompass the entire spectrum of pancreatic and periampullary diseases was based on the shared diagnostic and therapeutic pathways that require a multidisciplinary and highly specialized clinical approach.

### The Hub/Spoke operational model

The development of a national treatment network followed the principles outlined in the Bratislava Statement [19] and in the European Cancer Organisation Essential Requirements for Quality Cancer Care (ERQCC) [20] for improving PC care, according to which the referral centers should be formally or informally linked to providers with a different level of specialization. In line with these recommendations, the Steering Committee aimed to establish a coordinated network of treatment centers with varying degrees of expertise, ensuring an equitable distribution across regions according to their respective catchment areas.

vReferral centers were defined as multidisciplinary organizations capable of delivering standardized diagnostic and therapeutic pathways for pancreatic and periampullary diseases through a structured multidisciplinary approach. This framework led to the adoption of a Hub-and-Spoke operational model. Hub centers—identified as Pancreas Units—are designated as the facilities where all complex procedures, including pancreatic surgery and other high-complexity diagnostic or therapeutic procedures requiring advanced multidisciplinary expertise, should be concentrated, whereas Spoke centers function as satellite units, formally linked to the Hubs, and responsible for routine clinical activities and patient management.

An additional key component of the Steering Committee’s proposal concerns the training requirements for professionals working within Pancreas Units, outlining strategies for structuring dedicated educational programs from both logistical and administrative perspectives.

The categorization of centers into Hubs and Spokes was based on surgical adequacy metrics derived from Age.na.s data. Specifically, the Steering Committee endorsed the following criteria: (1) catchment area; (2) annual surgical volume; (3) ninety-day postoperative mortality rate; (4) long-term survival following surgical resection (3-year survival).

### Objective of the centralization model

- Clinical objectives: amelioration of survival and quality of life of patients affected by pancreatic and periampullary neoplasms through the shortening of diagnostic timeframes, prompt treatment of jaundice and/or duodenal obstruction, implementation of standardized diagnostic and treatment strategies, access to clinical trials, reduction of surgical risks and inappropriate surgical procedures, prompt activation of palliative care assistance and implementation of the surveillance of patients at risk of developing PC;
- Procedural objectives: implementation of the Hub/Spoke network, implementation of diagnostic and treatment strategies, collaboration with associations of patients against PC;
- Research objectives: to define research goals in a multicenter setting;
- Training objectives: to develop a training program between Hub and Spoke centers and implement educational programs.

## Results

### Minimum requirements for Hub centers (or pancreas units)

The Hub centers must present a multidisciplinary team and setting able to ensure the continuous availability of all required clinical specialties and services 365 days a year, 24 h a day. These requirements must be met within a single hospital.

The physical presence of all essential specialists and clinical services within a single institution is a non-negotiable quality and safety requirement for the designation of a center as Pancreas Unit. Specifically, the following clinical specialties and services must be present:

- Pancreatic surgery with an on-call service 24/7;
- Anesthesia and Intensive Care Units with proven experience in post-operative management of pancreatic surgery procedures available 24/7;
- Interventional Radiology Unit with proven experience in percutaneous and endovascular diagnosis and treatment of pancreatic and periampullary diseases as well as in the post-operative management of surgical complications available 24/7;
- Gastroenterology Unit with proven experience in the management of pancreatic and periampullary diseases;
- Diagnostic and Interventional Endoscopy Unit with proven experience in the perioperative management of pancreatic and periampullary diseases available 24/7;
- Oncology Unit with proven experience with proven experience in the management of pancreatic and periampullary neoplasms. Facilities for structured oncological follow-up should be an integral component of the Pancreas Unit, ensuring timely post-treatment surveillance, early detection of disease recurrence, prompt initiation of further oncological therapies, and continuity of care. Adequate follow-up pathways are essential to optimize long-term outcomes and may contribute to improved survival in patients with pancreatic and periampullary diseases;
- Radiotherapy Unit with proven experience in the management of pancreatic and periampullary neoplasms;
- Diagnostic Radiology Unit with proven experience in the diagnosis of pancreatic and periampullary diseases;
- Histopathologic Service with a frozen section analysis service available 12 h a day and proven experience in the diagnosis and molecular analysis of pancreatic and periampullary diseases;
- Medical Genetics Service;
- Nuclear Medicine Unit with proven experience in the diagnosis of pancreatic and periampullary diseases;
- Endocrinology Unit with proven experience in diabetes management;
- Pain Management Unit;
- Clinical Nutrition Service;
- Physiotherapy and Rehabilitation Service;
- Psychology Service;
- Geriatrics Service;
- Palliative Care Unit.

Moreover, the establishment of a dedicated outpatient clinic for the management and follow-up of pancreatic cystic lesions and neuroendocrine tumors is strongly recommended. Pancreatic surgical procedures must be performed exclusively in Hub centers. All clinical cases eligible for surgery should be reviewed during multidisciplinary team (MDT) meetings. Hub centers are also expected to collaborate with at least one patient association involved in pancreatic cancer advocacy. In addition, each Hub center must appoint a referral physician responsible for coordinating the MDT and its activities. If the referral physician is not a surgeon, the center must also designate a referral surgeon responsible for ensuring adherence to standardized surgical pathways from the preoperative to the postoperative phases.

Based on the catchment area analysis across Italian regions, one Hub center per 700,000–1,200,000 inhabitants is considered sufficient to meet population needs. The optimal number of Hub centers recommended by the Steering Committee for each region or autonomous province is reported in Table 1. While regions may identify a certain number of centers within their territory, the maximum number indicated in the table must not be exceeded.

**Table 1:** Optimal number of pancreas units for each region/autonomous province as recommended by the Steering Committee

Region/Autonomous Province	Population	Number of pancreatic resections per year	Number of centers to be established	Estimated number of pancreatic resections per center per year	Catchment area
Piemonte	4,341,375	329	4–5	66–82	1,100,000 − 850,000
Valle d’Aosta	126,933	19	0		
Lombardia	10,103,969	814	11	74	920,000
Trentino Alto Adige	1,074,819	70	2		500,000
Veneto	4,907,704	363	4–6	60–90	1,200,000 − 800,000
Friuli Venezia Giulia	1,211,357	88	1–2	44–88	1,200,000 − 600,000
Liguria	1,543,127	139	2	70	800,000
Emilia Romagna	4,467,118	403	4–6	68–100	1,100,000 − 750,000
Toscana	3,722,729	354	3–4	70–120	1,200,000 − 750,000
Umbria	880,285	72	1	72	880,000
Marche	1,518,400	103	1–2	50–100	1,500,000 − 750,000
Lazio	5,865,544	359	5–7	50–70	1,100,000 − 800,000
Abruzzo	1,305,770	90	1–2	45–90	1,300,000 − 650,000
Molise	308,493	20	0		
Campania	5,785,861	257	5–6	40–50	1,200,000 − 950,000
Puglia	4,008,296	223	4–5	45–55	1,000,000 − 800,000
Basilicata	556,934	28	1	28	550,000
Calabria	1,924,701	98	2	50	950,000
Sicilia	4,968,410	279	4–5	55–70	1,200,000 − 1,000,000
Sardegna	1,630,474	100	2	50	800,000

This network may be expanded and periodically renewed through dedicated healthcare training initiatives and must be integrated with the national research network [18]. Such integration is essential to ensure a translational approach and to support the sharing of expertise, infrastructure, and technological resources across the Hub-and-Spoke system.

### Spoke centers

Spoke centers are defined as active units that support and collaborate with their corresponding Hub center. They must adhere to the diagnostic and therapeutic pathways established at a national and regional level and operationally coordinated by the Hub center and are required to present clinical cases at the MDT meetings of the reference Hub.

Surgical procedures cannot be performed in Spoke centers; however, they must meet all non-surgical requirements expected of Hub centers. Specifically, Spoke centers are required to provide the following services: diagnostic and interventional radiology, medical and radiation oncology, clinical nutrition, and palliative care.

### MDT meetings

The MDT meetings represent a core element for the diagnostic and therapeutic pathway of patients and must be coordinated by the Hub center, through a nurse figure called Case Manager. The Case Manager is responsible for the: organization of the multidisciplinary meetings, collection of clinical cases to be discussed, verification of the medical reports according to a dedicated checklist (Online Resource 2), drafting the multidisciplinary decision for the final approval by the participants, organization of additional exams when required.

It is highly recommended the participation of at least one dedicated specialist per domain of the multidisciplinary group to the multidisciplinary meetings.

Cases followed by the Spoke centers must be presented at the MDT meetings of the reference Hub center through the presence of a Spoke center representative physician.

More complex cases, when needed, can be discussed at dedicated multidisciplinary meetings among Hub centers even remotely.

Multidisciplinary meetings in the Hub center must be held at least once a week. Multidisciplinary meetings between Hub and Spoke centers must be held at least once every two weeks.

As a whole, at least the 75% of all the cases and at least the 90% of surgical cases must be discussed at the multidisciplinary meetings.

Beyond frequency and case coverage, the effectiveness of MDT meetings is intended to be monitored through process-related indicators, including adherence to MDT decisions, time from diagnosis to treatment initiation, and compliance with standardized diagnostic–therapeutic pathways. The regional digital information platform will enable traceability of MDT recommendations and subsequent clinical actions, allowing progressive evaluation and refinement of MDT performance over time.

### Diagnostic and therapeutic pathway (DTP)

One of the key aspects of the Hub centers is the implementation and standardization of DTP for patients presenting with a suspected diagnosis of pancreatic or periampullary neoplasm. The healthcare staff (nursing or medical) are responsible for the assessment of patients’ need and completion of the diagnostic pathway. This must be accomplished through the standardization of the diagnostic and staging process, the assessment of the genetic/familial risk, the assessment of patients’ needs, the communication to the patients and relatives on the diagnostic steps and diagnostic hypotheses and proving them information on their rights.

This process is coordinated by the Case Manager.

All data of the DTP will be registered in a Regional Digital Information Platform for document sharing, coordination between Hub and Spoke centers and monitoring of clinical outcomes and process related to the management of patients. In practical terms, the Regional Digital Information Platform supports remote Hub–Spoke multidisciplinary meetings, secure sharing of imaging studies and clinical documentation, and tracking of referrals and key time points along the diagnostic–therapeutic pathway. This digital infrastructure facilitates coordinated decision-making, continuity of care, and timely access to specialized evaluation, and represents a potentially transferable organizational tool for other healthcare systems.

### Minimum requirements for each specialty within the Pancreas Unit (Hub Center) (Table 2)

See Table 2.

**Table 2:** Minimum requirements for the Multidisciplinary Team of a Pancreas Unit (Hub)

Specialist and clinical services	Minimum requirements
Surgery Unit with proven experience in pancreatic surgery, 24/7	*Volume criteria*: At least 30 pancreatic resections per year for benign/malignant pancreatic or periampullary neoplasms, with a 90-day mortality < 10%, aiming to reduce the 90-day mortality to below 5% within three years For mini-invasive surgery, at least 20 mini-invasive resections/year *Staff selection criteria*: One surgeon with experience of at least 60 pancreatoduodenectomy (PD) as first operator One surgeon with experience of at least 20 PD as first operator
Interventional Radiology Unit with proven experience, 24/7	*Staff selection criteria*: Two interventional radiologists with more than 5 years of experience as first operator in performing visceral angiograms, embolization, endovascular stent placement, percutaneous drain placement (biliary and/or other), biopsies and tumor ablations *Organizational criteria*: On call service
Diagnostic and Interventional Endoscopy, operational unit/service with proven experience, 24/7	*Staff selection criteria*: One endoscopist with proven experience in performing endoscopic ultrasound (> 250 procedures) and ERCP (> 300 procedures) For ERCP demonstration of success in papilla; cannulation > 80%; success in positioning biliary endoprostheses (distal stenosis) > 90%; incidence of clinically relevant pancreatitis post-ERCP < 10% *Organizational criteria*: Collaboration with the pathology department, including the possibility of rapid assessment of the adequacy of the biopsy sample Availability of spy-glass technology Possibility of duodenal stenting placing On call service
Oncology Unit with proven experience	*Volume criteria*: Fifty new patients/year treated for metastatic and non-metastatic neoplasms *Organizational criteria*: One oncologist in charge of pancreatic pathology Beds, DH service, and outpatient clinic available for pancreatic oncology Oncologist on call and booking center Organization of at least one training course/year focused on pancreatic cancer with Continuing Medical Education (ECM) certification
Radiotherapy Unit with proven experience	*Staff selection criteria*: Specific skills and training in the management of pancreatic disease demonstrable at curricular level including for example: continuity in training, participation in working groups and PDTA, contributions to the drafting of guidelines, conduct of research trials and publication of scientific works in the field of pancreatic cancer and presentations at national and international conferences *Technical criteria*: CT scan with 2–3 mm thickness slices acquisition, with custom-made immobilization system, with contrast in three-phase protocol and respiratory control system; 4D CT, respiratory gating, CT performed in free-breathing and breath-hold, abdominal compressor; contouring of Gross Tumor Volume (GTV), Clinical Target Volume (CTV), Planning Target Volume (PTV), Internal Target Volume (ITV), and Organ at Risk (OAR), and fusion of diagnostic RM and/or FDG PET scans with simulation CT; 3D Conformal Radiation Therapy, Intensity-Modulated Radiation Therapy, and Stereotactic Body Radiation therapy
Diagnostic Radiology Unit with proven experience	*Staff selection criteria*: Three radiologists with more than 10 years of experience in performing/reporting Ultrasound, CT, and MRI, elective and emergency examinations of pancreatic pathology Two Nuclear Medicine Physicians with more than 10 years of experience in performing/reporting PET/CT *Technical criteria*: Last generation CEUS, multistrato CT (minimum 64 detectors), MR (minimum 1.5 T), PET-CT, digital angiograph *Organizational criteria*: On call service
Histopathology services with documented experience in intraoperative frozen section analysis available for at least 12 h per day, rapid on-site evaluation (ROSE) of biopsies, and molecular pathology	*Staff selection criteria*: Pathologist with proven experience in extemporaneous and definitive histological and cytological diagnosis of pancreatic pathologies *Organizational criteria*: Molecular diagnostic laboratory for genotypic characterization Availability of a rapid assessment service for the adequacy of biopsy samples Analysis of surgical specimens according to international guidelines, with study of the circumferential resection margins at 1 mm Reporting times for the echo-endoscopic cyto-histological sample or first diagnosis within 5 days of the sample being acquired
Clinical Nutrition Service/Operative Unit	*Staff selection criteria*: Presence of medical staff (medical specialists in Food Science) and dietician (possibly also nutritional biologists to replace dieticians) within the facility *Organizational criteria*: Presence of codified pathways for multidisciplinary nutritional management for each patient who comes to the center’s observation from the moment of diagnosis Prescription and possibility of monitoring home artificial nutrition Access time for first visits no longer than 15 days in patients with a diagnosis already performed Possibility of carrying out/participating in research projects
Palliative Care Units	*Organizational criteria*: Coding of nutritional support pathway for patients with pancreatic neoplasms Prescription and possibility of monitoring home artificial nutrition (as per DDGW 14274 of 25/10/2021 and subsequent amendments) Access time for first visits no longer than 15 days in patients with a diagnosis already performed Possibility of carrying out/participating in research projects

#### Minimum requirements for pancreatic surgery

- At least 30 pancreatic resections per year for benign/malignant pancreatic or periampullary neoplasms, with a 90-day mortality < 10%, aiming to reduce the 90-day mortality to below 5% within three years. Hub centers with a mortality > 5% must formalize a strategy to achieve a reduction of 90-day mortality to < 5% in the next three years. In hospitals where multiple surgical departments are active, volume and outcome metrics must be attributed to each individual operational unit, rather than to the hospital as an aggregate. For this reason, it is recommended that a single operational unit be designated to admit and manage all patients undergoing pancreatic resections.
- At least two experienced pancreatic surgeons with the following criteria:
- one surgeon with a past experience of at least 60 pancreaticoduodenectomies as first operator.
- one surgeon with a past experience of at least 20 pancreaticoduodenectomies as first operator.

For performing minimally invasive pancreatic surgeries, surgeon must have performed at least 20 minimally invasive distal pancreatectomies and 20 minimally invasive pancreaticoduodenectomies.

#### Minimum requirements for medical oncology

Volume criteria: at least 50 new cases per year of metastatic and non-metastatic diseases treated by a dedicated oncology team.

Organizational criteria: availability of inpatients beds and outpatient centers for PC; organization of at least one training course per year on PC with a continuing medical education (ECM) certification; a referral oncologist for PC.

#### Minimum requirements for diagnostic and interventional radiology

Technical criteria: ultrasound machine, provided with harmonic imaging, color Doppler, capable to perform contrast enhanced ultrasound (C.E.U.S); multidetector computed tomography (CT) scan (minimum 64 detectors); magnetic resonance imaging (MRI) (minimum 1.5 Tesla); digital angiography; positron emission tomography (PET)-CT scanner.

Staff selection criteria: 3 radiologists with more than 10 years of experience in performing/reporting Ultrasound, CT, and MRI, elective and emergency examinations of pancreatic pathology; 2 nuclear medicine physicians with more than 10 years of experience in performing/reporting PET/CT; 2 interventional radiology with more than 5 years of experience as first operator in performing splanchnic angiographies, embolization procedures, endovascular stent placements, percutaneous drainage placement, biopsies and ablations.

Organizational criteria: on-call service for radiology and interventional radiology.

#### Minimum requirements for diagnostic and interventional digestive endoscopy and gastroenterology

Staff selection criteria: one endoscopist with proven experience in performing endoscopic ultrasound (> 250 procedures) and Endoscopic retrograde cholangiopancreatography (ERCP) (> 300 procedures); for ERCP, demonstration of clinical success in papilla cannulation > 80%; success in positioning biliary endoprostheses (distal stenosis) > 90%; incidence of clinically relevant pancreatitis post-ERCP < 10%.

Organizational criteria: collaboration with the pathology department, including the possibility of rapid assessment of the adequacy of the biopsy sample; availability of spy-glass technology; possibility of duodenal stenting placing; on-call service.

#### Minimum requirements for cytological and hystopatological and molecular diagnostic

Staff selection criteria: pathologist with proven experience in extemporaneous and definitive histological and cytological diagnosis of pancreatic pathologies.

Organizational criteria: molecular diagnostic laboratory for genotypic characterization; availability of a rapid assessment service for the adequacy of biopsy samples; analysis of surgical specimens according to international guidelines, with study of the circumferential resection margins at 1 mm; reporting times for the echoendoscopic cyto-histological sample or first diagnosis within 5 days of the sample being acquired.

#### Minimum requirements for radiotherapy

Staff selection criteria: specific skills and training in the management of pancreatic disease demonstrable at curricular level including continuity in training, participation in working groups and DTP, contributions to the drafting of guidelines, conduct of research trials and publication of scientific works in the field of pancreatic and periampullary diseases and presentations at national and international conferences.

Technical criteria: CT scan with 2–3 mm thickness slices acquisition, with custom-made immobilization system, with contrast in three-phase protocol and respiratory control system; 4D CT, respiratory gating, CT performed in free-breathing and breath-hold, abdominal compressor; contouring of Gross Tumor Volume (GTV), Clinical Target Volume (CTV), Planning Target Volume (PTV), Internal Target Volume (ITV), and Organ at Risk (OAR), and fusion of diagnostic RM and/or FDG PET scans with simulation CT; 3D Conformal Radiation Therapy, Intensity-Modulated Radiation Therapy, and Stereotactic Body Radiation therapy.

#### Minimum requirements for palliative care

Organizational criteria: prescription and possibility of monitoring home artificial nutrition; access time for first visits no longer than 15 days in patients with a diagnosis already performed; possibility of carrying out/participating in research projects.

#### Minimum requirements for nutrition service

Staff selection criteria: presence of medical staff (medical specialists in Food Science) and dietician (possibly also nutritional biologists to replace dieticians) within the facility.

Organizational criteria: presence of codified pathways for multidisciplinary nutritional management for each patient who comes to the center’s observation from the moment of diagnosis; prescription and possibility of monitoring home artificial nutrition; access time for first visits no longer than 15 days in patients with a diagnosis already performed; possibility of carrying out/participating in research projects.

#### Research

From a research perspective, one of the priority objectives in establishing a national system of Pancreas Units should be the development of a national pancreatic and periampullary diseases research network. Through structured interaction with the national preclinical and translational research network, clinical investigators conducting independent clinical trials would benefit from more rapid scientific exchange with basic and translational scientists. This collaboration would facilitate the analysis of collected biological samples, with the aim of generating new scientific hypotheses, exploring innovative therapeutic combinations, and developing novel biomarkers.

To foster a virtuous cycle between preclinical and clinical research, the proposed independent national clinical research network for pancreatic and periampullary diseases in Italy should also contribute to:

- Supporting the technological transfer of new experimental therapies into clinical testing, including the design and conduct of early-phase clinical trials (phase I/II);
- Establishing a national biobank network containing tumor samples and matched normal tissues collected from patients enrolled in clinical studies, with structured access procedures for Italian preclinical researchers within the preclinical/translational network. This infrastructure would allow laboratory validation of the molecular and cellular mechanisms underlying sensitivity or resistance to investigational treatments, support the generation of new scientific hypotheses, and enable the development of innovative biomarkers for precision pancreatic oncology.

## Discussion

PC is characterized by a rising incidence and persistently poor survival outcomes. Epidemiological projections indicate that both incidence and mortality will increase sharply over the next 30 years (+ 34% and + 37%, respectively, in men and women) [21], underscoring the urgency of strengthening national strategies for its management.

Given the complexity of this disease—requiring timely diagnosis, coordinated multidisciplinary input, and access to highly specialized therapeutic and supportive services—the establishment of referral centers dedicated to pancreatic and periampullary diseases represents a critical step toward improving the quality, consistency, and equity of care for one of the most challenging malignancies in modern oncology. In this context, the Steering Committee’s proposal to implement a national network of Pancreas Units responds to an urgent clinical, organizational, and ethical need, aligning Italy with international best practices and addressing long-standing disparities in access to care.

A fundamental premise of the Steering Committee is that every patient, regardless of regional origin or socioeconomic background, has the right to receive comparable standards of care. Yet, historical data from the Age.na.s underscore significant regional inequalities: during 2014–2016, approximately 40% of patients residing in Southern Italy—and up to 76% in specific territories—migrated to Northern regions for pancreatic surgery, while 15% of patients from Central Italy did the same [16]. More recent data reported by Age.na.s confirm the persistence of this phenomenon. Notably, regional escape indices reached 100% in some regions, such as Molise and Valle d’Aosta in 2022, indicating the absence of locally available surgical pathways and reinforcing the need for a coordinated national framework aimed at balancing centralization with equitable territorial access [16].

This large-scale cross-regional mobility highlights both a lack of uniformly distributed expertise and the consequent burden placed on patients, families, and the healthcare system. Travel-related limitations may disproportionately affect the elderly, socioeconomically disadvantaged individuals, and those with severe comorbidities, further worsening pre-existing inequities in access to high-quality care.

The Steering Committee therefore adopted a centralizing strategy based on a Hub-and-Spoke architecture, aimed at distributing pancreatic care in a rational manner across the national territory. Hub centers, or first-level Pancreas Units, are envisioned as highly specialized institutions able to provide continuous, multidisciplinary, and technologically advanced care 24 h a day, 7 days a week. Spoke centers, or second-level referral sites, support the Hubs by managing diagnostic pathways, oncology care, palliative interventions, and follow-up, while adhering to the same standardized protocols and participating actively in MDT meetings. Together, these centers form an integrated network designed to ensure timely referral, reduce inappropriate or suboptimal treatments, and enable high-volume hubs to concentrate the most complex surgical and interventional procedures.

International experience strongly supports this direction. The Netherlands represents a well-established example of successful centralization: since 2011, efforts by the Dutch Pancreatic Cancer Group to consolidate pancreatic surgery in a limited number of high-volume centers have led to significant reductions in operative mortality and major postoperative complications [22–24]. Germany has adopted a certification system through the German Cancer Society, requiring Pancreas Centers to meet defined structural, procedural, and outcome-related benchmarks [25, 26]. Other European countries, including Norway and the UK, have embraced similar models, further validating the principle that complex pancreatic procedures should be centralized in dedicated, high-volume facilities [27–29]. Even in the United States—where healthcare regulation differs substantially—initiatives such as the Leapfrog Group’s recommendations and the “Take the Volume Pledge” campaign have attempted to guide patients toward high-volume institutions, recognizing that volume correlates strongly with outcomes, regardless of the system’s structure [30]. It should be acknowledged that no universally optimal surgical volume threshold for pancreatic resections exists, and that international policies reflect substantial heterogeneity in this regard. The proposed minimum volume of ≥ 30 pancreatic resections per year should therefore be interpreted as a pragmatic, policy-driven compromise between ideal volume–outcome standards and national feasibility. This threshold was defined to ensure adequate geographic coverage while promoting progressive quality improvement, rather than as an absolute or optimal cutoff applicable across different healthcare systems.

While certain components of the proposed model reflect the specific organization of the Italian National Health Service, the availability of national outcome registries, and centralized public governance, the core principles of the Pancreas Unit network are broadly applicable across different healthcare systems. These include multidisciplinary management, centralization of complex procedures in high-performance centers, standardized diagnostic–therapeutic pathways, structured Hub–Spoke networks, and progressive monitoring of quality and outcomes. Accordingly, the proposed framework should be interpreted as an adaptable model rather than a rigid blueprint, allowing tailoring to local healthcare structures and resources.

The evidence supporting centralization is robust: global studies consistently show that hospitals performing low annual volumes of pancreatic resections have significantly higher operative mortality rates [12–14]. In Italy, observational studies confirm the same trend: centers performing five or fewer pancreatic resections annually report mortality as high as 12.4%, compared with 2.6% in centers performing 90–100 procedures [15]. Importantly, surgical expertise alone does not fully explain these outcomes. Failure-to-rescue—that is, the inability of a hospital to effectively manage postoperative complications—is a major determinant of mortality after pancreatic surgery. High-volume hospitals typically possess the full spectrum of interventional radiology, endoscopy, angiography, critical care, and advanced nursing capabilities required to manage severe postoperative events. These institutional factors are indispensable to ensuring acceptable outcomes and cannot be replicated in low-volume, poorly equipped regional hospitals.

A unique aspect of the national Italian model is its attention not only to surgical centralization but also to the organization of diagnostic and therapeutic endoscopy. Many advanced procedures—such as EUS-guided fine-needle biopsy, EUS-guided biliary drainage, and EUS-guided gastroenterostomy—are highly operator-dependent and currently performed in only a limited number of centers with adequate case volume and expertise. Nationwide analyses from other European regions show significant variability in diagnostic accuracy for EUS-guided tissue acquisition, with sensitivity for malignancy as low as 65% in low-volume centers [31]. Similarly, ERCP performance varies widely, prompting the development of quality measures and benchmarks. Evidence indicates that ERCP performed in high-volume centers yields higher clinical success and lower complication rates at lower cost, strongly supporting its inclusion among the procedures recommended for centralization [32]. Ensuring that patients have access to high-performance endoscopy is critical: delayed diagnosis or suboptimal palliation—when due to inadequate endoscopic expertise—can significantly compromise therapeutic pathways and patient survival.

The Steering Committee’s work also emphasizes the crucial role of non-medical professionals and the necessity of integrating them into the care model. Nurse case managers, dietitians, psycho-oncologists, physiotherapists, and palliative care specialists contribute to different phases of the patient journey, ensuring both clinical and psychosocial support. Nurse case managers serve as essential facilitators of multidisciplinary discussion and care continuity. Dietitians play a central role in preventing malnutrition, a key determinant of postoperative morbidity in pancreatic and periampullary diseases. Physiotherapists support prehabilitation and rehabilitation, mitigating surgical stress and improving functional recovery. These components reflect the shift toward holistic, patient-centered care, which is an essential philosophical and operational foundation of Pancreas Units.

Training and workforce preparation constitute another core element. The Steering Committee recognizes that in several regions, the lack of specialized expertise represents a major barrier to implementing a national network. For this reason, the proposed model includes structured training programs in which clinicians undergo a progressive pathway of competency acquisition through mentoring, tutoring, and proctoring in high-volume centers. This approach mirrors successful models adopted internationally and ensures that the development of Pancreas Units is not limited solely to structural requirements but includes human capital formation, essential for long-term sustainability.

To ensure equitable access to specialized care, the proposed model includes an initial three-year development phase, during which regions are allowed to progress toward meeting the required criteria for Hub designation. With respect to training and integration, the proposed model is designed to enable progressive competency development through structured collaboration between Hub and Spoke centers, including mentoring, proctoring, and shared clinical pathways. The integration of Spoke centers, particularly in geographically remote areas, is supported by standardized diagnostic–therapeutic pathways, digital platforms, and remote participation in multidisciplinary team meetings, aiming to reduce geographic barriers while maintaining centralized decision-making and quality standards.

This phased implementation recognizes regional differences in hospital capabilities, professional expertise, and healthcare organization, while still committing to national uniformity in standards. Hubs are required to fulfill tightly defined criteria—including minimum surgical volume (at least 30 pancreatic resections per year for benign/malignant pancreatic or periampullary neoplasms, with a 90-day mortality < 10%, aiming to reduce the 90-day mortality to below 5% within three years. Hub centers with a mortality > 5% must formalize a strategy to achieve a reduction of 90-day mortality to < 5% in the next three years). They must also provide full multidisciplinary teams with 24/7 availability in surgical, anesthesiology, radiology, endoscopy, oncology, pathology, genetics, nutrition, rehabilitation, psychology, geriatrics, and palliative care.

Spoke centers play an equally important role in the network. While they do not perform pancreatic surgery, they must adhere to the diagnostic–therapeutic pathway established by the Hub, participate in multidisciplinary meetings, and provide key services such as diagnostic imaging, oncology and radiotherapy, nutrition, and palliative care. The operational coordination between Hubs and Spokes ensures that patients are evaluated promptly and appropriately, with complex decisions centralized but supportive care delivered close to home, balancing the need for specialization with accessibility.

Despite the clear advantages of centralization, certain challenges remain. Geographic barriers, uneven distribution of resources, variations in hospital infrastructure, and differences in regional health governance may affect implementation. These challenges can be mitigated by strengthening inter-regional collaboration, enhancing telemedicine, and implementing standardized referral systems. Continued monitoring of outcomes and quality indicators will also be essential to evaluate the impact of the Pancreas Units model and refine its structure over time.

## Conclusions

Overall, the national initiative to establish Pancreas Units represents a robust and forward-looking strategy to improve pancreatic and periampullary diseases care in Italy. By integrating multidisciplinary expertise, centralizing complex procedures in high-performing centers, defining minimum structural and organizational requirements, and investing in structured training programs, the proposed model provides a strong foundation for enhancing patient outcomes and reducing variability in care. Furthermore, its structured yet adaptable design may serve as a reference model for other countries seeking to improve the management of these complex and resource-intensive diseases.

## Supplementary Information

Below is the link to the electronic supplementary material.

Supplementary Material 1

## Data Availability

The datasets generated during and/or analysed during the current study are available from the corresponding author on reasonable request.
