# Supplementary material for: Designing a national network of pancreas units: the Italian model for high-quality pancreatic cancer care
Source: Updates Surg. 2026 Mar 24;78(3):1219–31. doi: 10.1007/s13304-026-02598-7 (PMC13249644; doi:10.1007/s13304-026-02598-7)

**Journal:** Updates in Surgery

**Designing a National Network of Pancreas Units: The Italian Model for High-Quality Pancreatic Cancer Care**

Sergio Alfieri^1,2,3^, MD; Giuseppe Quero^1,2,3^, MD, PhD; Vincenzo Tondolo^2,4^, MD; Giampaolo Balzano^5^, MD; Antonella Cardone^6^, MD; Giovanni Conzo^7^, MD; Carlo Fabbri^8^, MD; Viviana Ferrari^9^; Luca Frulloni^10^, MD, PhD; Salvatore Gruttadauria^11,12^, MD; Elisabetta Ianelli^13^, JD; Evaristo Maiello^14^, MD; Roberta Menghi^1,2,3^, MD; Michele Milella^15^, MD, PhD; Michele Reni^16^, MD; Roberto Salvia^17^, MD, PhD; Cristiano Spada^2,18^, MD, PhD; Manuela Tamburo De Bella^19^, MD; Ilaria Tarantino^20^, MD; Giampaolo Tortora^2,21^, MD, PhD; Federica Valsecchi^22^, PhD, MBA; Leonardo Vincenti^23^, MD; Fabio Vistoli^24,25^, MD, PhD; Ugo Boggi^26^, MD, PhD;

^1^ Pancreatic Surgery Unit, Fondazione Policlinico Universitario “Agostino Gemelli” IRCCS, Rome, Italy

^2^ Dipartimento di Scienze Mediche e Chirurgiche, Università Cattolica del Sacro Cuore di Roma, Rome, Italy

^3^ Gemelli Pancreatic Center, CRMPG (Advanced Pancreatic Research Center) Fondazione Policlinico Universitario “Agostino Gemelli” IRCCS, Rome, Italy

^4^ General Surgery Unit, Fatebenefratelli Isola Tiberina—Gemelli Isola, Rome, Italy

^5^ Division of Pancreatic Surgery – Circolo di Varese Hospital, Varese, Italy

^6^ Cancer Patients Europe, Brussels, Belgium

^7^ Division of General and Oncological Surgery, Department of Translational Medical Sciences University of Campania 'Luigi Vanvitelli', Naples, Italy

^8^ Digestive Endoscopy and Gastroenterology Unit, Forlì-Cesena Hospitals, Azienda Unita Sanitaria Locale della Romagna, Forlì-Cesena, Italy

^9^ Nastro Viola Association

^10^ Department of Medicine, Gastroenterology and Digestive Endoscopy, University of Verona, Verona, Italy

^11^ Department for the Treatment and Study of Abdominal Diseases and Abdominal Transplantation, Istituto Di Ricovero E Cura a Carattere Scientifico-Istituto Mediterraneo Per I Trapianti E Terapie Ad Alta Specializzazione (IRCCS-ISMETT), Palermo, Italy

^12^ Department of Surgery and Medical and Surgical Specialties, University of Catania, Catania, Italy

^13^ Italian Federation of Volunteer-based Cancer Organizations (FAVO), Rome, Italy

^14^ Medical Oncology Unit, Fondazione IRCCS “Casa Sollievo della Sofferenza”, San Giovanni Rotondo, Italy

^15^ Section of Innovation Biomedicine-Oncology Area, Department of Engineering for Innovation Medicine, University of Verona, and Verona University and Hospital Trust, Verona, Italy.

^16^ Department of Medical Oncology, Pancreas Translational and Clinical Research Center, IRCCS San Raffaele Scientific Institute, Milan, Italy

^17^ Department of Pancreatic Surgery - Azienda Ospedaliera Universitaria Verona, Verona, Italy

^18^ Digestive Endoscopy Unit, Fondazione Policlinico Universitario Agostino Gemelli IRCCS, Rome, Italy

^19^ Hospital Clinical Networks Governance e DM70/15 Monitoring—AGENAS—National Agency for Regional Health Services (Age.na.s)

^20^ Digestive Endoscopy Service, Department of Diagnostic and Therapeutic Services, IRCCS - ISMETT, Palermo, Italy

^21^ Medical Oncology, Comprehensive Cancer Center, Fondazione Policlinico Universitario "A Gemelli"- IRCCS, Rome, Italy

^22^ Nadia Valsecchi Foundation

^23^ Unit of Surgery, Department of Surgery Sciences, National Institute of Gastroenterology "S. de Bellis", IRCCS Research Hospital, Castellana Grotte, Bari, Italy

^24^ San Salvatore Hospital ASL1 Abruzzo, Division of General and Transplant Surgery, L'Aquila, Italy

^25^ University of L'Aquila, Department of Biotechnological and Applied Clinical Sciences, L'Aquila, Italy

^26^ Division of General and Transplant Surgery, University of Pisa, Pisa, Italy

**Corresponding author:**

Giuseppe Quero

Pancreatic Surgery Unit

Department of Surgery, Fondazione Policlinico Universitario “Agostino Gemelli”, IRCCS

Largo Agostino Gemelli, 8

00168 Rome, Italy

tel.: +39 06 30 15 51 33

fax: +39 06 30 15 65 20

e-mail : [giuseppe.quero@policlinicogemelli.it](mailto:giuseppe.quero@policlinicogemelli.it)

| **Online Resource 1. Patient Mobility Indicators and Economic Impact by Italian Region** | | | | | | | | |
| --- | --- | --- | --- | --- | --- | --- | --- | --- |
| **Region** | **Outflow^1^** | **Inflow^2^** | **Net Balance^3^** | **Outflow Index^4^ (%)** | **Inflow Index^5^(%)** | **Revenue^6^ (€)** | **Cost^7^ (€)** | **Economic Balance^8^ (€)** |
| **Southern Italy** |  |  |  |  |  |  |  |  |
| Abruzzo | 28 | 3 | -25 | 60.87 | 14.29 | 37,416 | 365,146 | -327,730 |
| Basilicata | 17 | 1 | -16 | 89.47 | 33.33 | 9,558 | 210,567 | -201,009 |
| Puglia | 49 | 7 | -42 | 32.67 | 6.86 | 84,390 | 606,371 | -521,981 |
| Molise | 12 | 1 | -11 | 100 | 7.14 | 13,929 | 158,406 | -144,477 |
| Calabria | 36 | 0 | -36 | 85.71 | 0 | 0 € | 475,218 | -475,218 |
| Campania | 68 | 1 | -67 | 39.53 | 39.53 | 13,929 | 881,621 | -867,692 |
| **Central Italy** |  |  |  |  |  |  |  |  |
| Lazio | 40 | 42 | 2 | 17.09 | 40.38 | 550,064 | 650,151 | -100,087 |
| Marche | 41 | 8 | -33 | 66.13 | 3.33 | 89,577 | 509,760 | -420,183 |
| Toscana | 23 | 39 | 16 | 10.65 | 4.70 | 652,090 | 307,254 | 344,836 |
| Umbria | 28 | 2 | -26 | 73.68 | 16.67 | 27,858 | 350,673 | -322,815 |
| **Northen Italy** |  |  |  |  |  |  |  |  |
| Emilia-Romagna | 60 | 20 | -40 | 26.43 | 10.70 | 265,467 | 825,848 | -560,381 |
| Friuli-Venezia Giulia | 18 | 1 | -17 | 32.73 | 2.63 | 9,558 | 224,496 | -214,938 |
| Liguria | 28 | 1 | -27 | 38.89 | 2.22 | 13,929 | 374,999 | -361,070 |
| Lombardia | 37 | 154 | 117 | 9.95 | 31.11 | 2,055,853 | 509,621 | 1,546,232 |
| Autonomous Province of Bolzano | 2 | 0 | -2 | 8.33 | 5.33 | 0 | 29,623 | -29,623 |
| Autonomous Province of Trento | 14 | 0 | -14 | 53.85% | 5.67 | 0 | 188,735 | -188,735 |
| Piemonte | 33 | 8 | -25 | 19.88 | 6.80 | 102,690 | 424,689 | -321,999 |
| Sardegna | 29 | 0 | -29 | 43.28 | 11.11 | 0 | 386,405 | -386,405 |
| Sicilia | 71 | 0 | -71 | 41.52 | 12.68 | 0 | 998,562 | -998,562 |
| Valle d’Aosta | 4 | 0 | -4 | 100.00 | 0 | 0 | 46,974 | -46,974 |
| Veneto | 5 | 355 | 350 | 1.97 | 58.77 | 4,655,343 | 56,532 | 4,598,811 |

^1^Outflow: number of patients residing in a given region who received pancreatic surgical care in a different region; ^2^Inflow: number of patients residing outside the region who received pancreatic surgical care within that region; ^3^Net Balance: difference between inflow and outflow (Inflow – Outflow), expressing the region’s net patient mobility; ^4^Outflow Index (%): Proportion of residents who sought pancreatic surgical treatment outside their region of residence, calculated as Outflow / (Outflow + Inflow) × 100; ^5^Inflow Index (%): proportion of patients treated in the region who came from other regions, calculated as Inflow / (Outflow + Inflow) × 100; ^6^Revenue (€): total financial reimbursement received by the region for treating non-resident patients, based on national DRG tariffs for pancreatic and periampullary procedures; ^7^Cost (€):total financial expenditure incurred by the region for residents treated outside the region, corresponding to DRG reimbursements paid to the hosting region; ^8^Economic Balance (€): net financial balance derived from patient mobility, calculated as Revenue – Cost.

Online Resource 2 - Sample multidisciplinary team (MDT) checklist used for case preparation and documentation prior to discussion
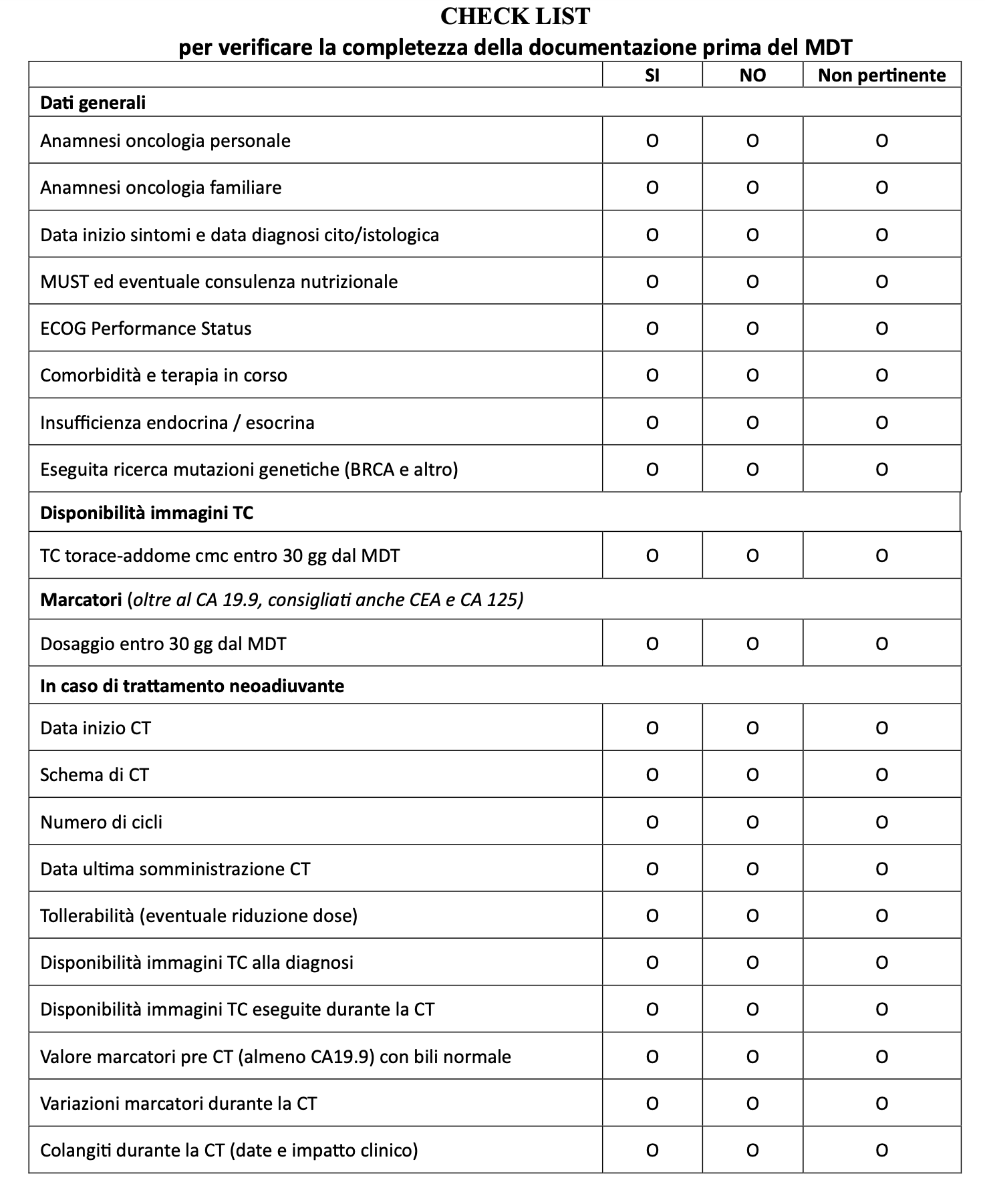

Supplement: Supplementary file 1 — Supplementary Material 1 [file 13304_2026_2598_MOESM1_ESM.docx]
